# Cross-species Study of Canine and Human Peripheral Nerve Sheath Tumors: Clinical and Molecular Perspectives

**DOI:** 10.64898/2026.07.28.740600

**Published:** 2026-07-29

**Authors:** Jace P. Landry, Angela D. Bhalla, Sharon M. Landers, Rossana Lazcano, Lindsay A. Parker, Tasha M. Miller, Noelle Niemi, Heather Lyu, Heather Lillemoe, Emily Z. Keung, Christopher P. Scally, Christina L. Roland, Kelly K. Hunt, John M. Slopis, Ian E. McCutcheon, Beth Boudreau, Heather Wilson-Robles, Alexander J. Lazar, Kunal Rai, Dominique J. Wiener, Brian W. Davis, Brandan Wustefeld-Janssens, Keila E. Torres

## Abstract

Malignant peripheral nerve sheath tumors (MPNSTs) are aggressive sarcomas. An obstacle to treating MPNSTs is a lack of effective systemic therapies. Although over 70% of human MPNSTs have lost or inactivated the epigenome regulator polycomb repressive complex 2 ( *PRC2* ), its activity and contribution to canine PNST progression remain unclear. This study compared canine peripheral nerve sheath tumors (PNSTs) and human MPNSTs across biological and clinical features, including PRC2 activity. Immunohistochemical analysis was performed for a human tissue microarray of 54 neurofibromas and 139 MPNSTs, and 63 canine PNSTs for H3K27me3, a repressive histone mark deposited by intact PRC2, and H3K27ac, which increases globally upon H3K27me3 loss. To understand the genomic alterations present in canine PNSTs, we analyzed tumor mutation burden, copy number alteration, and transcriptomes of eight canine PNST/normal pairs. The results suggested that H3K27me3 loss and associated gain of H3K27ac epigenetically drive human and canine tumors. These findings warrant further studies to evaluate whether these epigenetic deregulations alter similar gene signatures across species.

## Full Text Availability

The license terms selected by the author(s) for this preprint version do not permit archiving in PMC. The full text is available from the preprint server.

